# Impact of Covid-19 on Psychosocial Well-Being of School-Going Children: A Cross-Sectional Study

**DOI:** 10.7759/cureus.62561

**Published:** 2024-06-17

**Authors:** Deepti Damayanty Pradhan, Pravati Jena, Sreesom Misra, Bijay Kumar Meher, Leena Das

**Affiliations:** 1 Pediatrics, Kalinga Institute of Medical Sciences, KIIT University, Bhubaneswar, IND; 2 Pediatrics, Sriram Chandra Bhanja Medical College and Hospital, Cuttack, IND; 3 Pediatrics, Post Graduate Institute of Medical Education and Research and Capital Hospital, Bhubaneswar, IND

**Keywords:** sars-cov-2, hope, emotions, conduct disorder, attention deficit hyperactivity disorder

## Abstract

Introduction: The mandated closure of schools due to Covid-19 is likely to have a negative impact on school-going children. This study aimed to assess the psychosocial well-being of school children during the pandemic in eastern India.

Methods: This cross-sectional study was conducted in the outpatient pediatric department of tertiary care teaching hospitals. Children between the ages of 4 and 14 were enrolled. The main outcome measures included the Emotional Symptoms Scale, Conduct Problem Scale, Hyperactivity Scale, Peer Problem Scale, and Prosocial Scale from the Strength and Difficulties Questionnaire (SDQ), as well as the Children’s Hope Scale.

Results: Out of 169 children aged 4-14, 104 (61.5%) were male, 140 (82.8%) were from urban areas, 66 (39.1%) had a family member who was a healthcare worker or frontline worker, and 12 (7.1%) had experienced the death of a family member due to Covid-19. Anxiety-related and depressive symptoms were observed in 81 (47.9%) and 70 (41.4%) children, respectively. Psychosocial difficulties with a 'clinically significant problem likely' were observed in 26 (15.4%) children, more common in males (16.35%, P=0.035) and older children (12-14 years). Children from families with healthcare/frontline workers, Covid-affected families, loss of job in the earning member, and uninvolved parenting style were associated with more psychosocial difficulties. The mean (SD) hope score was 22.46 ± 6.42 in children above eight years.

Conclusion: The psychosocial well-being of school-going children is adversely affected during Covid-19, particularly in families with frontline workers, loss of job, and death of family members due to Covid-19. The poor hope score in children aged 8 years and above indicates an adverse impact on their ability to achieve future goals.

## Introduction

Social distancing was one of the primary public health interventions to reduce the spread of SARS-CoV-2, the virus-causing Covid-19. Though children appeared to be at low risk of developing severe disease and mortality, many interventions (closure of school, quarantine of family members, news and social media on Covid-19, separation of parents, death of near and dear ones, etc.) have significantly disrupted the lives of children [1]. Past evidence has shown significant psychosocial effects on children during disasters, and reports of psychosocial distress in children have increased during Covid-19 [2-4]. The core components of psychological well-being are emotional/personal, social/interpersonal and the ability to cope. During the initial crisis period, the 'emotional' and 'social' components are likely to be affected whereas during the protracted and recovery phase, the 'ability to cope' component is affected.

The objective of the present study was to describe the various components of psychosocial well-being of school-going children during the pandemic and their associated factors. Psychosocial components included are depression, anxiety, behaviour, emotion, conduct, socialisation, and goal-oriented thought.

## Materials and methods

This survey was conducted from June 2021 to December 2021 after receiving ethical approval from the Institutional Ethical Committee, Balangir. The parents of school-going children aged between 4 and 14 years were invited to participate in the study either in person or via WhatsApp, email, and URL-linked messages. After obtaining informed written consent, a pre-designed questionnaire was distributed to the parents. Children over the age of eight were asked to provide their assent to participate in the study. Children not attending school or known to have psychosocial, neuropsychiatric, or developmental disorders were excluded from the study.

 The questionnaires were given to parents, who were encouraged to seek clarification if they didn't understand any items when scoring. Clarifications were given by the administrator by making the parents understand the item in local language or citing examples relevant to the question. No compensation was provided to the participants. The study aims and outcomes were not disclosed to the participants to avoid response bias. The parents were asked to involve family members and children aged eight or above to complete the survey together. Triangulation of data was encouraged to ensure the best response. Incomplete or missing data and inappropriate case selections were excluded from the analysis.

Survey instrument

The questionnaire includes identification and demographic information, five selected questions from the child depression scale, four questions from the anxiety scale, 33 questions from the Strength and Difficulties Questionnaire (SDQ), and six questions from the Children's Hope Scale [5,6]. The SDQ can be downloaded for free from http://www.sdqinfo.org. It comprises five subscales, each with five items, measuring emotional symptoms, conduct problems, hyperactivity-inattention, peer relationship problems, and prosocial behaviors [5]. The demographic section includes questions about age, sex, residence, parental occupation (whether a parent is a healthcare/frontline worker or not), and any loss of household income or death of a family member due to Covid-19. The items were selected from the Parent Report Measures for Children and Adolescents SDQ (P) 04-10 and Parent Report Measures for Children and Adolescents SDQ (P) 11-17. Six items from the Children's Hope Scale were included in the questionnaire as self-report questions for children aged 8-14. The collected data were analyzed.

The first 25 SDQ items were grouped into five scales: emotional symptoms scale, conduct problem scale, hyperactivity scale, peer problem scale, and prosocial scale. Each response was converted into a score (0 for 'Not true', 1 for 'Somewhat true', and 2 for 'Certainly true'). For items 07, 11, 14, 21, and 25, a reverse scoring was applied (i.e., 2 for 'Not true', 1 for 'Somewhat true', and 0 for 'Certainly true'). The total difficulty score was calculated as:

$Total\ Score = Emotional\ Scale + Conduct\ Scale + Hyperactivity\ Scale + Peer\ Problem\ Scale$ 

The interpretation of the SDQ score was based on the information from its website and classified as ‘Average’, 'Slightly raised', and 'High'. If the score is average, it means that a clinically significant problem in the area is unlikely. If the score is slightly raised, it indicates that a clinically significant problem is likely. If the score is high, there is a substantial risk of a clinically significant problem.

The Children's Hope Scale was administered to children above eight years old and labeled as 'Questions about your goals'. The total Children's Hope Scale score was achieved by adding the responses to the six items: 'None of the time' 1; ‘A little of the time' 2; 'Some of the time' 3; 'A Lot of the time' 4; 'Most of the time' 5; 'All of the time' 6.

Sample size

Assuming a 12% prevalence of psychosocial problems during the pandemic and a precision of 5% with 95% confidence interval, we calculated a minimum sample size of 163. However, we gathered data from 169 subjects, which exceeds the required minimum sample size. 

Statistical analysis

The data were analyzed using SPSS version 24.00. Descriptive statistics were used to analyze the descriptive data for both continuous and categorical variables. Categorical variables were presented as the number and percentage of patients and were compared, if necessary, using Pearson’s chi-square test for independence of attributes or Fisher's exact test. Continuous variables were presented as mean and standard deviation and compared using unpaired t-test or analysis of variance (ANOVA). The association of psychosocial factors with different demographic parameters and styles of parenting was examined using Pearson’s correlation coefficient. A difference was considered statistically significant when the p-value was less than 0.05.

## Results

Out of 205 questionnaires that were distributed, 22 couldn't be collected and 14 were incomplete. A total of 169 questionnaires were completed and analyzed. Table 1 displays the baseline characteristics of the study population. Out of 169 school-going children, there were more males than females, with a ratio of 1.6:1. 140 (82.8%) of the children lived in urban areas, 129 (76.3%) came from families with an employed adult, and 66 (39.1%) had a frontline worker or healthcare worker in the family. During Covid-19, 14 (8.3%) families had earning members who lost jobs, 56 (33.1%) family members were affected by the pandemic, and 12 (7.1%) succumbed to the virus. Authoritative and permissive parenting styles were observed in 117 (69.2%) and 29 (17.2%) families, respectively.

**Table 1:** Baseline characteristics of school-going children in the study population (n=169) Data presented as n (%).

Characteristics						No (%)
Age						
	4-8 Years					65 (39.2)
	>8-11 Years					40 (24.1)
	>11-14 Years					61 (36.7)
Gender						
	Male					104 (61.5)
	Female					65 (38.5)
Residence						
	Urban					140 (82.8)
	Rural					29 (17.2)
Employed adult in family						
		Yes				129 (76.3)
		No				40 (23.7)
Healthcare/frontline worker in family						
			Yes			66 (39.1)
			No			103 (60.9)
Loss of job in earning family member						
				Yes		14 (8.3)
				No		155 (91.7)
Family member affected with Covid-19						
				Yes		56 (33.1)
				No		113 (66.9)
Death of family member due to Covid-19						
					Yes	12 (7.1)
					No	157 (92.9)
Parenting style						
					Uninvolved	16 (9.5)
					Permissive	29 (17.2)
					Authoritarian	7 (4.1)
					Authoritative	117 (69.2)

Anxiety-related symptoms and depressive symptoms were found in 70 (41.4%) and 81 (47.9%) school-going children in the study population. Psychosocial problem was assessed by total difficulties score in SDQ scale. Clinically significant problems in total difficulty scores were found in 26 (15.4%) children. Clinically significant problems in emotion, conduct, hyperactivity, peer relation and socialisation were found in 13 (7.7%), 39 (23.1%), 40 (23.6%), 68 (40.2%), 41 (24.2%) children respectively. Goal of children was assessed by Children’s Hope scale. In school-going children in the age group of 8-14 years, the mean (SD) hope score was 22.46 ± 6.42. (Table 2).

**Table 2:** Psychosocial problems and Hope Score of school-going children in the study population Data presented as n (%), * mean (SD)

Problems	Overall (n=169)
Depressive symptoms	81 (47.9)
Anxiety-related symptoms	70 (41.4)
Overall psychosocial difficulties	26 (15.4)
Clinically significant emotional symptoms	13 (7.7)
Clinically significant conduct problem	39 (23.1)
Clinically significant hyperactivity symptoms	40 (23.6)
Clinically significant peer problem	68 (40.2)
Clinically significant socialization problem	41 (24.2)
*Hope Score (n=132)	22.46 (6.42)

Table 3 shows the socio-demographic factors associated with anxiety and depressive symptoms in school-going children during Covid-19. Anxiety-related and depressive symptoms were more commonly seen in children from urban areas, families with healthcare/frontline workers, Covid-19-affected families, loss of jobs in the earning member of the family, and uninvolved parenting styles. Anxiety-related symptoms were more common in females, with 30 (46.15%) of them affected, and in older age groups (12-14 years), with 29 (47.54%) affected. However, depressive symptoms were more common in males, with 51 (49.04%) affected, and in younger children (4-8 years), with 34 (52.3%) affected.

**Table 3:** Socio-demographic factors associated with anxiety and depressive symptoms in the study population Data presented as n (%)

Variable	Anxiety-related symptoms (n=70)	P value	Depressive symptoms (n=81)	P value
					Age				
4-8 Years	25 (38.4)	0.475	34 (52.3)	0.657
9-11 Years	16 (37.21)		19 (44.19)	
12-14 Years	29 (47.54)		28 (45.9)	
Sex				
Male	40 (38.46)	0.323	51 (49.04)	0.715
Female	30 (46.15)		30 (46.15)	
Residence				
Urban	63 (45)	0.038	69 (49.29)	0.438
Rural	7 (24.14)		12 (41.38)	
Employed adult member in family				
Yes	53 (41.09)	0.874	66 (51.16)	0.131
No	17 (42.5)		15 (37.5)	
Healthcare/frontline worker in family				
Yes	29 (43.94)	0.595	35 (53.03)	0.288
No	41 (39.81)		46 (44.66)	
Family member affected in Covid-19				
Yes	25 (44.64)	0.549	31 (55.36)	0.174
No	45 (39.82)		50 (44.25)	
Loss of job in family				
Yes	8 (57.14)	0.212	10 (71.43)	0.066
No	62 (40.0)		71 (45.81)	
Family member death in Covid-19				
Yes	5 (41.67)	0.986	4 (33.33)	0.294
No	65 (41.4)		77 (49.04)	
Parenting style				
Uninvolved	9 (56.25)	0.348	10 (62.5)	0.341
Permissive	9 (31.03)		16 (55.17)	
Authoritarian	2 (28.57)		2 (28.57)	
Authoritative	50 (42.74)		53 (45.3)	

In Table 4, we can see the psychosocial challenges and the average hope score from the SDQ questionnaire and Children’s Hope Scale. The research indicates that older children (12-14 years old), city dwellers, families affected by Covid-19, those who have experienced job loss in the family, or those with uninvolved parenting styles are more likely to experience psychosocial difficulties. Additionally, the average Hope Score was lower in children who have experienced the death of a family member and those with permissive parenting styles.

**Table 4:** Socio-demographic factors associated with clinically significant psychosocial difficulties and Hope Score in the study population Data presented as n (%)

Variable	Psychosocial difficulties (n=70)	P value	*Hope Score (n=132)	P value
Age				
4-8 Years	6 (9.2)	0.213	23.20 (7.54)	0.480
9-11 Years	8 (18.6)		20.97 (6.62)	
12-14 Years	12 (19.6)		22.76 (5.24)	
Sex				
Male	17 (16.35)	0.661	22.33 (6.59)	0.771
Female	9 (13.85)		2.67 (6.21)	
Residence				
Urban	24 (17.14)	0.164	22.24 (6.37)	0.401
Rural	2 (6.9)		23.46 (6.65)	
Employed adult member in family				
Yes	19 (14.73)	0.671	22.29 (6.4)	0.557
No	7 (17.5)		23.11 (6.58)	
Healthcare/frontline worker in family				
Yes	6 (9.1)	0.069	23.00 (6.75)	0.444
No	20 (19.42)		22.11 (6.21)	
Family member affected in Covid-19				
Yes	9 (16.07)	0.862	23.07 (5.38)	0.456
No	17 (15.04)		22.17 (6.87)	
Loss of job in family				
Yes	4 (28.57)	0.153	21.46 (6.70)	0.556
No	22 (14.2)		22.57 (6.40)	
Family member death in Covid-19				
Yes	2 (16.67)	0.898	19.73 (6.57)	0.140
No	24 (15.28)		22.71 (6.37)	
Parenting style				
Uninvolved	3 (18.75)	0.855	22.46 (5.11)	0.089
Permissive	3 (10.35)		19.52 (7.88)	
Authoritarian	1 (14.29)		21.57 (6.80)	
Authoritative	19 (16.24)		23.31 (5.99)	

## Discussion

The present study was conducted on school-going children during Covid-19. The children mostly belonged to urban areas, the majority having an employed adult in the family, and many were from families with frontline/healthcare workers. School closure, lack of outdoor activity, and aberrant dietary and sleeping habits have disrupted the children’s usual lifestyle. Children of frontline/healthcare workers have suffered unique problems [7]. Loss of a job in a family member, separation of a family member for being quarantined due to infection or job or death of any family member has significantly affected the psychosocial well-being of the school-going children. The mental health status of many children has been affected manifested by anxiety and depressive symptoms with reduced hope score in older children.

We evaluated the mental health status of children using anxiety and depressive symptoms. Anxiety- and depression-related symptoms are found in around 45% of children. Anxiety-related symptoms are reported to increase during lockdown as compared to before lockdown in Dutch children and adolescents [8]. Zainudeen et al. have reported 28.5% of children with anxiety, and 31.4% with depression in Malaysian families using the Depression Anxiety and Stress Scale (DASS) [9]. Duan et al. have reported the anxiety level of children to be 23.87±15.79 (Spence Child Anxiety Scale); 22.28% suffering from depressive symptoms (Child Depression Inventory) during the Covid-19 outbreak [3]. Healthy children as well as children with special healthcare needs have a high prevalence (57.4%) of mental health problems during Covid-19 in several studies [10,11].

Older children and females have more anxiety symptoms whereas younger children and males have more depressive symptoms. Saddik et al. have reported a high prevalence (59.8%) of anxiety in young people during Covid-19 with females having 1.91 times higher odds of reporting anxiety than males [12]. However, loss of household income and being female is associated with higher odds of depressive and anxiety symptoms in school-going children from Florida during Covid-19 [13].

We have used SDQ for assessing the psychosocial difficulties and reported them as clinically significant problems likely. Conduct, hyperactivity and socialization problems were seen in around 24% of children. Emotional problems were seen in 7.7% but peer relation problems in 40% of children. Using the SDQ scale, in Chinese adolescents the prevalence of emotional and behavioral problems was higher (31.6% for total difficulties score and 37.5% for prosocial problems) as compared to the school-going children in our cohort [14]. Zainudeen et al. have reported that 38% of children with high psychosocial impact (score 14) in Malaysian families using the Impact of Event Scale-Revised (IES-R) and Children’s Revised Impact of Event Scale (CRIES) [9]. In a cross-sectional study of Hong Kong families, the risk of psychosocial problems was higher in children with special educational needs, acute/chronic disease, single-parent families and low-income families [15]. In a study from UAE, 17.5% of school-aged children had emotional problems [12]. Children from urban areas have fewer opportunities for interaction due to the stringent implementation of lockdown measures as compared to rural areas. We found more anxiety, depressive symptoms as well as psychosocial difficulties in urban children. In a study by Schneiderman et al. from Argentina during the Covid-19 lockdown, 96.3% of parents noticed emotional changes in their children. Boredom, irritability, and reluctance were more present during lockdown [16].

Anxiety and depressive symptoms were found in school-going children during pandemic and more so with those belonging to the family with healthcare or frontline workers. Psychosocial difficulties (emotional, prosocial, hyperactivity, peer relation and conduct problems) are lower in families with a frontline/healthcare worker as compared to those not having a frontline/healthcare worker.

Family is a protective factor against psychosocial difficulties especially at the time of lockdown and school closure when children don’t have the opportunity to physically meet their friends. Family harmony gets disturbed when any earning family member loses a job, gets infected with Covid-19, or dies of Covid-19. We found more anxiety and depressive symptoms and psychosocial difficulties in the children from disturbed families. Moulin et al. reported elevated levels of ADHD and emotional symptoms in children from families with financial difficulties [17]. Luijten et al. have reported worse mental/social health in children from families with a friend/relative infected with Covid-19 or negative change in work situation due to Covid-19 regulations [18]. On the other hand, an increased number of child abuse cases were reported during lockdown. National Crime Record Bureau in India estimated that around 40,810 children fell victim to sexual offences, and in 95% of cases, the perpetrator was known to the victim under regular circumstances before the pandemic [18]. However, during the lockdown 50% increase in call rates to CHILDLINE India with around 30% reporting child abuse was noted [19]. Poor mental health, unemployment and frustration of parents during the pandemic with the lack of a defense system (school teachers, friends) might be the reasons for the same [20].

Parenting style has an impact on the development of children. Uninvolved parenting style was associated with more anxiety, depressive symptoms, and psychosocial difficulties in our cohort. Authoritarian parenting was protective for psychosocial impact during Covid-19. Parenting under pressure had a mixed impact on family life during Covid-19, providing an opportunity to teach health concerns, and hygiene to kids, balancing the act of parenting (assisting with children’s schoolwork and working from home), and improving family relationships and parent-child bonding [21].

In the present study, it was observed that psychosocial problems in school-going children are significant during Covid-19. Poor hope score in children (≥8 years) indicates an adverse impact on achieving future goals and can have devastating consequences if not addressed properly. Peer problems and socialization problems will adversely affect the social life of children and can affect their mental health leading to depression, anxiety disorders and substance abuse [22]. Children with significant conduct problems are at risk of developing other mental disorders such as anti-social personality disorder, mood or anxiety disorders, violence, juvenile delinquency, suicidal and criminal behavior [23]. Children with significant emotional problems are at risk of developing anxiety, major depressive disorder and suicidal behavior [24]. Hyperactivity may be a sign of ADHD. A decline in the mean hope score indicates that more attention needs to be given to the children in the form of proper parenting and guidance by teachers. Early addresal of these issues can improve the psycho-social well-being of the children before it’s too late.

The strength of the present study needs special mention. Gathering data from the parents on psychosocial aspect of school-going children especially during lockdown and school closure was difficult. However, it has several limitations too. The majority of the respondents belonged to urban areas, and many had an employed parents in the family. Sample size is small and does not represent the entire population of the state. However, the impact of the pandemic seems to be universal and applicable to all children of school going age. The psychosocial problems of the population prior to the pandemic are not available. Prospective studies that include evaluation of anxiety and depression post pandemic/lockdown in follow up can give a better representation of the magnitude of the problem. Further study with implementation of psychosocial intervention will be useful adjunct to the present findings.

## Conclusions

Covid-19, along with mandated lockdowns and school closures, has had a negative impact on the mental and emotional well-being of school-going children. This impact is especially pronounced in families with frontline workers, job loss, and the death of family members due to the pandemic. Anxiety-related symptoms are more prevalent in female and older children, while depressive symptoms are more common in male and younger children. Children aged 8 and above with a low hope score may experience a negative effect on their ability to achieve future goals. These findings suggest that policymakers should develop effective coping strategies for school-going children. Schools should prioritize screening strategies, implement mental health interventions based on significant influencing factors, and focus on supporting the vulnerable group.

## Appendices

**Table 5:** Psychosocial well-being of school-going children SDQ: Strength and Difficulties Questionnaire

Psychosocial well-being of school-going children						
Personal details :						
1	Email Id :					
2	Name of the child :					
3	Age in months :					
4	Date of birth :					
5	Sex :	Male			Female	
6	Phone No. :					
7	Address (District, State, country) :					
8	Residence :	Rural			Urban	
9	Studying in which class /standard :					
10	Name of school :					
11	Is any adult member at your home currently employed ?	Yes			No	
12	Is anyone in your family a health care/front line worker ?	Yes			No	
13	Does any earning family member lost job during Covid-19 ?	Yes			No	
14	Does any family member affected with Covid-19?	Affected in the 1st wave		Affected in the 2nd wave		Not affected
15	Is any family member succumbed to Covid-19?	Yes			No	
Past illness and medications						
Please mention if your child is suffering from any chronic diseases and/or regular medications						
16	Seasonal allergies :	Yes			No	
17	Heart disease :	Yes			No	
18	Liver disease :	Yes			No	
19	Kidney disease :	Yes			No	
20	Immunosupression :	Yes			No	
21	Others (If any, specify) :					
22	Any regular medications (If any, specify) :					
Changes in behavior and parenting						
Please mark the change in behavior which you have noticed in your baby during this pandemic which was not present before. Tick one or more options.						
23	1) Have you noticed your baby :	Eating poorly/not feeling hungry				
		Not sleeping well				
		Not happy/more quiet/crying frequently/too tired				
		None of the above				
24	2) Have you noticed your baby :	Worried too much/can't stop worrying about Cvodi-19				
		Feeling afraid of getting infected with Covid-19				
		Scared of being alone/scared of insects/ darkness				
		None of the above				
25	3) Is your baby :	Not attending online classes				
		Watching TV/mobile all the time				
		developed nail biting/thumb sucking/bed wetting				
		Engaged in arts/drawings/dance/other activities				
		Playing indoor games (ludo/carrom/ chess/ others)				
		Following a regular routine of class/play/ entertainment/other activities				
26	4) On a Sunday, you and your daughter both are tired. Your daughter has homework and announces she needs lots of help, despite your throbbing headache. What you say…	I will help you, but get started on it on your own and do what you can.				
		It's not my homework. You have to do it. Do it or else you will be punished.				
		Why do I do your homework with you?				
		I have such a headache. Please do it on your own or just skip it for tonight, after all you are also tired.				
27	5) If your child asks you to let him/her go to a place which you don't like, how do you respond ?	I strictly say no. I don't let him argue on those topics which I don't like.				
		I say 'no' and explain him why he shouldn't go there. If he argues, I listen to him and make him understand why he is wrong.				
		I let him go and keep a track of him till he returns home.				
		I always fulfil his demands. I never interfere in his day today activities.				
Strength and difficulties questions						
For each item, please mark the most appropriate box. Choose the best one you think even if you are not absolutely certain. Please give your answers on the basis of your child's behavior over the last six months.						
28	1) Considerate of other people's feelings (SDQ PS1)				Not true	
					Somewhat true	
					Certainly true	
29	2) Restless, overactive, cannot stay still for long (SDQ H1)				Not true	
					Somewhat true	
					Certainly true	
30	3) Often complains of headaches, stomach-aches or sickness (SDQ E1)				Not true	
					Somewhat true	
					Certainly true	
31	4) Shares readily with other children, for example toys, treats, pencils (SDQ PS2)				Not true	
					Somewhat true	
					Certainly true	
32	5) Often loses temper (SDQ CPS1)				Not true	
					Somewhat true	
					Certainly true	
33	6) Rather solitary, prefers to play alone (SDQ PPS1)				Not true	
					Somewhat true	
					Certainly true	
34	7) Generally well behaved, usually does what adults request (SDQ CPS2)				Not true	
					Somewhat true	
					Certainly true	
35	8) Many worries or often seems worried (SDQ E2)				Not true	
					Somewhat true	
					Certainly true	
36	9) Helpful if someone is hurt, upset or feeling ill (SDQ PS3)				Not true	
					Somewhat true	
					Certainly true	
37	10) Constantly fidgeting or squirming (SDQ H2)				Not true	
					Somewhat true	
					Certainly true	
38	11) Has at least one good friend (SDQ PPS2)				Not true	
					Somewhat true	
					Certainly true	
39	12) Often fights with other children or bullies them (SDQ CPS3)				Not true	
					Somewhat true	
					Certainly true	
40	13) Often unhappy, depressed or tearful (SDQ E3)				Not true	
					Somewhat true	
					Certainly true	
41	14) Generally liked by other children (SDQ PPS3)				Not true	
					Somewhat true	
					Certainly true	
42	15) Easily distracted, concentration wanders (SDQ H3)				Not true	
					Somewhat true	
					Certainly true	
43	16) Nervous or clingy in new situations, easily loses confidence (SDQ E4)				Not true	
					Somewhat true	
					Certainly true	
44	17) Kind to younger children (SDQ PS4)				Not true	
					Somewhat true	
					Certainly true	
45	18) Often lies or cheats (SDQ CPS4)				Not true	
					Somewhat true	
					Certainly true	
46	19) Picked on or bullied by other children (SDQ PPS4)				Not true	
					Somewhat true	
					Certainly true	
47	20) Often volunteers to help others (parents, teachers, other children) (SDQ PS5)				Not true	
					Somewhat true	
					Certainly true	
48	21) Thinks things out before acting (SDQ H4)				Not true	
					Somewhat true	
					Certainly true	
49	22) Steals from home, school or elsewhere (SDQ CPS5)				Not true	
					Somewhat true	
					Certainly true	
50	23) Gets along better with adults than with other children (SDQ PPS5)				Not true	
					Somewhat true	
					Certainly true	
51	24) Many fears, easily scared (SDQ E5)				Not true	
					Somewhat true	
					Certainly true	
52	25) Good attention span, sees chores or homework through to the end (SDQ H5)				Not true	
					Somewhat true	
					Certainly true	
Complaints of teacher/caregiver						
Over the last six months, have your child’s teachers/caregiver complained of the following :						
53	1) Fidgetiness, restlessness or overactivity				No	
					A little	
					A lot	
54	2) Poor concentration or being easily distracted				No	
					A little	
					A lot	
55	3) Acting without thinking, frequently butting in, or not waiting for his or her turn				No	
					A little	
					A lot	
Difficulties in different areas						
Answer the first question, if answer is 'Yes', answer rest 7 questions						
56	Overall, do you think that your child has difficulties in any of the following areas: emotions, concentration, behavior or being able to get along with other people ?				No	
					Yes, minor difficulties	
					Yes, definite difficulties	
					Yes, severe difficulties	
57	How long have these difficulties been present ?				<1month	
					1-5 month	
					6-12 month	
					over 1year	
58	Do the difficulties upset or distress your child?				Not at all	
					A little	
					A medium amount	
					A great deal	
59	Do the difficulties interfere with your child’s everyday life in the HOME LIFE ?				Not at all	
					A little	
					A medium amount	
					A great deal	
60	Do the difficulties interfere with your child’s everyday life in the FRIENDSHIPS ?				Not at all	
					A little	
					A medium amount	
					A great deal	
61	Do the difficulties interfere with your child’s everyday life in the CLASSROOM LEARNING ?				Not at all	
					A little	
					A medium amount	
					A great deal	
62	Do the difficulties interfere with your child’s everyday life in the LEISURE ACTIVITIES ?				Not at all	
					A little	
					A medium amount	
					A great deal	
63	Do the difficulties put a burden on you or the family as a whole ?				Not at all	
					A little	
					A medium amount	
					A great deal	
Questions about your goal						
ONLY FOR CHILDREN AT/ABOVE 8 YEARS. Please mark the most appropriate answer by asking the following six questions regarding goal of your kid.						
64	1) I think, I am doing pretty well.				None of the time	
					A little of the time	
					Some of the time	
					A lot of the time	
					Most of the time	
					All of the time	
65	2) I can think of many ways to get the things in life that are most important to me.				None of the time	
					A little of the time	
					Some of the time	
					A lot of the time	
					Most of the time	
					All of the time	
66	3) I am doing just as well as other kids of my age.				None of the time	
					A little of the time	
					Some of the time	
					A lot of the time	
					Most of the time	
					All of the time	
67	4) When I have a problem, I can come up with lots of ways to solve it.				None of the time	
					A little of the time	
					Some of the time	
					A lot of the time	
					Most of the time	
					All of the time	
68	5) I think the things I have done in the past will help me in the future.				None of the time	
					A little of the time	
					Some of the time	
					A lot of the time	
					Most of the time	
					All of the time	
69	6) Even when others want to quit, I know that I can find ways to solve the problem.				None of the time	
					A little of the time	
					Some of the time	
					A lot of the time	
					Most of the time	
					All of the time	
Consent						
I do hereby give my consent to use the information submitted by me for analysis, which might help the researchers to find the psycho-social well-being and help the stakeholders to plan for psychosocial support measures for school going children.						
		Yes	No	Signature :		
